# Clinical description and development of a prognostic score for neurofibromatosis type 1 (NF1)-associated GISTs: a retrospective study from the NETSARC+☆

**DOI:** 10.1016/j.esmoop.2025.104477

**Published:** 2025-03-04

**Authors:** C. Cuvelier, M. Brahmi, I. Sobhani, B. Verret, A. Grancher, N. Penel, M. Toulmonde, W. Lahlou, H. Dupuis, L. Calavas, M. Muller, S. Watson, D. Bruyat, F. Poumeaud, L. Chaigneau, S. Manfredi, T. Lecomte, F. Bertucci, F. Ghiringhelli, M. Pracht, F. Mourthadhoi, L. Monceau-Baroux, M. Helyon, J.-E. Kurtz, G. Roquin, N. Regenet, M. Vinches, D. Tougeron, P. Wolkenstein, J.Y. Blay, O. Bouche, V. Hautefeuille

**Affiliations:** 1 Department of Gastroenterology and Digestive Oncology, Amiens University Hospital, Amiens, France; 2 Department of Medical Oncology, Centre Leon Berard, Lyon, France; 3 Department of Gastroenterology, Henri Mondor Hospital, Créteil, France; 4 Department of Medical Oncology, Gustave Roussy Cancer Campus, Paris Saclay University, Villejuif, France; 5 Department of Gastroenterology and Digestive Oncology, Rouen University Hospital, Rouen, France; 6 Department of Medical Oncology, Centre Oscar-Lambret, ULR-2694 - METRICS: Evaluation des technologies de santé et des pratiques médicales, CHU de Lille, University of Lille, Lille, France; 7 Department of Medical Oncology, Bergonié Institute, Bordeaux, France; 8 Department of Gastroenterology and Digestive Oncology, European Georges Pompidou Hospital, AP-HP, Paris-Cité University, Paris, France; 9 Department of Endocrinology, Diabetology, Metabolism and Nutrition, Huriez Hospital, Lille University Hospital, Lille, France; 10 Department of Gastroenterology and Digestive Oncology, Hospices Civils de Lyon, Lyon University Hospital, Lyon, France; 11 Department of Gastroenterology and Digestive Oncology, Nancy University Hospital, Vandoeuvre-lès-Nancy, France; 12 Department of Medical Oncology and INSERM U830, Institut Curie, Paris, France; 13 Department of Medical Oncology, Institut Paoli-Calmettes, Marseille, France; 14 Department of Medical Oncology, Oncopole Claudius Regaud, Toulouse, France; 15 Department of Medical Oncology, University Hospital of Besançon Jean Minjoz, Besançon, France; 16 Gastroenterology and Digestive Oncology Unit, University Hospital Dijon-Bourgogne, University of Burgundy, INSERM U1231, Dijon, France; 17 Department of Hepatogastroenterology and Digestive Oncology, Trousseau Hospital, Tours, France; 18 INSERM UMR 1069, Tours University, Tours, France; 19 Department of Medical Oncology, Institut Paoli-Calmettes, Aix-Marseille Université, Marseille, France; 20 Department of Medical Oncology, Georges François Leclerc, Dijon, France; 21 Department of Medical Oncology, Eugène Marquis Center, Rennes, France; 22 Department of Digestive Surgery and Oncology, CHU Saint-Etienne, Saint-Priest-en-Jarez, France; 23 Department of Medical Oncology, CHRU Brest Morvan Hospital, Brest, France; 24 Department of Digestive Surgery, Clermont Ferrand University Hospital, Clermont-Ferrand, France; 25 ICANS Cancer Institute, Strasbourg, France; 26 Gastroenterology and Digestive Oncology, Angers University Hospital, Angers, France; 27 Department of Digestive Surgery, Nantes University Hospital, Nantes, France; 28 Department of Medical Oncology, Cancer Institute of Montpellier, Montpellier, France; 29 Department of Gastroenterology and Hepatology, Poitiers University Hospital, Poitiers, France; 30 Department of Dermatology, Henri Mondor Hospital, Créteil, France; 31 Department of Gastroenterology and Digestive Oncology, CHU Reims, University Reims Champagne Ardennes, Reims, France

**Keywords:** neurofibromatosis type 1 (NF1), gastrointestinal stromal tumor (GIST), mitotic count, recurrence-free survival (RFS), adjuvant treatment, imatinib

## Abstract

**Background:**

Gastrointestinal stromal tumors (GISTs) occur in ∼7% of neurofibromatosis type 1 (NF1) patients. Data about their natural history remain scarce and neither risk classifications, prognosis model nor adjuvant treatment have been validated in this population.

**Methods:**

This national retrospective study included consecutive operated NF1-GIST cases from 31 reference centers in France, mostly from the NETSARC+ network. Factors associated with relapse were used to build a new prognostic score (RECKGIST). To address potential bias between adjuvant group and follow-up group, a propensity score was used.

**Results:**

A total of 119 patients were included between 2008 and 2023, of whom 61% were women. Median age was 53 years (range 20-78 years). The main primary location was the small bowel (86%) and the stomach (11%). Median size and mitotic count (mit) were 45 mm [95% confidence interval (CI) 45-58 mm] and 2 mit/5 mm^2^ (95% CI 3-9 mit/5 mm^2^), respectively. The vast majority were *KIT/PDGFRA* wild type (mutation *KIT* 2%, *PDGFRA* 3%). The median follow-up was 6 years. For GISTs <30 mm (*n* = 35), none relapsed. For GISTS >30 mm (*n* = 84), 18 developed metastases (21%). There was no difference in relapse according to tumor location (*P* = 0.45) or tumor rupture (*P* = 0.11), whereas *KIT/PDGFRA*-mutated GISTs were at higher risk of relapse [recurrence-free survival (RFS) at 10 years of 30% versus 82.5% for wild type, *P* = 0.03]. Miettinen and Joensuu classification did not predict relapse accurately. For the RECKGIST score A (size ≤30 mm, *n* = 34) group, 10-year RFS was 100%; it was 78.5% in the RECKGIST B group (size >30 mm and 0 < mit ≤ 5, *n* = 60), and 45.5% in the RECKGIST C group (size >30 mm and mit >5, *n* = 20) (*P* < 0.0001). After matching, 10-year RFS was similar between adjuvant and surveillance groups (*P* = 0.34).

**Conclusions:**

For NF1-GISTs <30 mm, prognosis without relapse is excellent. RECKGIST score accurately predicts recurrence and needs to be validated in an external cohort, but it may help treatment decision making. No efficacy of adjuvant treatment was observed.

## Highlights

• NF1-GISTs are mostly located in the small bowel but 5% of them are in the stomach.
• A *KIT/PDGFRA* mutation is harbored by 5% of NF1-GISTs, leading to a poorer prognosis (10-year RFS 30% versus 82.5%).
• Prognosis without relapse is excellent in GISTs <3 cm and this may define a new cut-off for surgery for asymptomatic tumors.
• RECKGIST score classifies accurately NF1-GISTs into three relapse groups, contrary to Miettinen and Joensuu classification.
• Adjuvant treatment has no purpose in NF1-GIST, as RFS after matching is similar between imatinib and surveillance groups.

## Introduction

Gastrointestinal stromal tumors (GISTs) are rare tumors of the digestive tract that arise from the mesenchymal tissue and belong to the sarcoma group. They may involve any part of the digestive tract but are frequently located in the stomach (60%-70%) and the small bowel (20%-30%) in the general population.^1^ Although usually sporadic, GISTs may be associated with a constitutive mutation, such as Carney-Stratakis dyad.^2^ More frequently, GISTs may be associated with Von Recklinghausen disease, also called neurofibromatosis type 1 (NF1), and occur in ∼7% of NF1 patients.^3^ NF1 is one of the most frequent genetic disorders in the European population, with a prevalence of ∼1/3000, and has an autosomal dominant transmission. In 50% of cases, it occurs without familial history of the disease (50% of cases with *de novo* mutation).^4^ Mutation of the *NF1* gene leads to an altered synthesis of neurofibromin, a protein that regulates the MAP kinase and the PI3K-AKT-mTOR pathways, inducing activation of the oncogenic pathways and development of several tumors.^5^

Diagnosis of NF1 is based on National Institutes of Health (NIH) criteria with at least two of the following present: café-au-lait macules, neurofibromas, Lisch nodules, freckling, optic gliomas, bony lesions and familial history of NF1.^6^ Genetic testing of the *NF1* gene is not mandatory but is encouraged. Expected survival of NF1 patients is shorter than the general population in Western countries with median age at death of 65 years compared with ∼80-90 years.^7^ Lifetime risk of cancer is higher than in the general population (59.6% compared with 30.8%),^8^ and the main causes of tumor-related death are malignant peripheral nerve sheath tumors (MPNST, 42%), central nervous system tumors (17%) and breast cancer (7%). Interestingly, GISTs in NF1 patients have a high 5-year survival rate (92%) compared with other malignancies in NF1 patients (5-year survival of 23% for high grade gliomas, 31% for MPNST and up to 67% for melanomas).^9^

NF1-GISTs represent 1.5% of all GISTs. Patient age at diagnosis is lower than in sporadic GISTs (50 years compared with 60 years), and there is a slight female predominance.^10^ Multiple tumors are found in 35%-62% of cases and are mostly located in the small bowel (89%), but may be found in the stomach (5%). They are often found incidentally (52%), but bleeding, anemia, abdominal pain or intestinal obstruction are not uncommon. In published cohorts, the primary tumor size is typically small (3-4 cm) with a low mitotic count (3 mit/5 mm^2^).^11^^,^^12^ The recurrence rate, however, is not well established (11% in the cohort reported by Miettinen et al.) and robust data from large cohorts with long follow-up are needed to better characterize these tumors at clinical, histological, molecular and prognostic levels. There has been no publication about the *NF1* mutation harbored by these GISTs. Finally, the value of usual risk classifications [i.e. the Armed Force Institute of Pathology (AFIP) classification of Miettinen et al. and the modified NIH classification of Joensuu et al.]^13^, ^14^, ^15^, ^16^ and the relevance of adjuvant imatinib therapy remain unknown.

The main objectives of our present study were to determine clinical, histological and molecular characteristics of NF1-GISTs; to determine the factors associated with relapse; to assess the relevance of AFIP/NIH classification in this population; and, if indicated, to build a new scoring system based on prognostic factors. The impact of adjuvant imatinib was also studied. We chose to focus on GISTs in Von Recklinghausen disease and to exclude sporadic GISTs with an *NF1* mutation but without evidence of the genetic disease, to avoid confusion bias and homogenize our study population.

## Patients and methods

### Study population

This retrospective multicentric study included all consecutive patients diagnosed with a histologically proven nonmetastatic GIST undergoing surgery, with a medical history of NF1 according to clinical NIH criteria and familial history of the disease or genetic testing for *NF1* mutation. GISTs with *NF1* somatic mutation but without evidence of Von Recklinghausen disease, metastatic GISTs at diagnosis and GISTs not treated surgically were excluded. Patients were recruited from 2008 to 2023 in 30 tertiary centers in France through the NETSARC+ and university hospital networks.

Clinical data, immunohistochemistry (such as KIT/CD117, DOG1, CD56, SDH), histopathological features [primary tumor size, mitotic count/5 mm^2^, Ki67 (%), tumor rupture], and somatic mutations identified through routine next-generation sequencing (NGS; including *RAS*, *BRAF* and *NF1* only for later patients), were collected for the largest GIST of each patient. Both French and ESMO European guidelines recommend *KIT/PDGFRA* mutation analysis. However, extended NGS analysis for other genes is not mandatory. Relapse was diagnosed based on results of imaging follow-up [i.e. computed tomography scan or magnetic resonance imaging].

The study was conducted in accordance with the Declaration of Helsinki and French regulatory requirements (*Commission Nationale Informatique et Libertés* registration number: 2218839, MR004 methodology). All patients alive at the time of the study received appropriate verbal information and gave their consent for anonymous data collection. In accordance with the French national laws and clinical research guidelines, this retrospective observational study did not require formal ethics committee approval.

### Objectives and statistical analysis

The primary objective was to determine the factors associated with relapse. The secondary objectives were to determine the relapse rate, recurrence-free survival (RFS) and overall survival (OS), and the capacity of usual risk classification (Miettinen and Joensuu classifications) to predict relapse. In the case of low ability of the classifications in predicting relapse, a new classification score was to be developed. The last secondary objective was to analyze the relevance of adjuvant treatment to determine its eventual impact on relapse. To address potential bias, 1 : 1 propensity scoring and inverse probability of treatment weighting (IPTW) matching were carried out between the adjuvant group (AG) and the surveillance group (SG).

Continuous variables were expressed as median and range and compared using the Student’s *t*-test and the Mann–Whitney *U* test. Categorical variables were expressed as percentages and total number and compared using the chi-square and Fisher’s exact tests. OS was calculated from date of diagnosis to death, and RFS from the date of the surgery to the date of relapse. Patients were censored at the date of last follow-up if no recurrence occurred. Survival analysis was carried out using the Kaplan–Meier method, expressed as median with 95% confidence interval (CI). The log-rank test was used for survival comparison between groups.

For all tests, *P* < 0.05 was considered statistically significant. Univariate analysis was carried out to assess factors associated with RFS. A factor with a *P* value <0.1 in univariate analysis was integrated in the multivariate analysis. Hazard ratios (HRs) and 95% CIs were used for estimation. All statistical analyses were carried out using EasyMedStat software version 3.34.2 (www.easymedstat.com).

## Results

### Population characteristics

A total of 138 patients with NF1-GIST as defined were identified. Of these, 19 were excluded: 9 had synchronous metastatic disease at diagnosis and 10 had a localized GIST but without surgery. Characteristics of the 119 included patients are summarized in Table 1. All patients had a confirmed diagnosis of Von Recklinghausen disease according to the NIH criteria.

**Table 1:** Baseline characteristics of the RECKGIST cohort

Clinical characteristics	*n* (%)	Relapse, *n* (%) (*N* = 18)	No relapse, *n* (%) (*N* = 101)	*P*
Female	73 (61.3)	11 (61.1)	62 (61.4)	0.99
Median age at diagnosis, year (range)	53 (20-78)	57 (26-74)	51 (19-78)	0.06
Symptomatic patients with GIST complications at diagnosis, including:	52 (43.7)	12 (66.7)	40 (39.6)	0.08
Digestive hemorrhage	20 (16.8)	3	17	
Digestive obstruction	6 (5.0)	3	3	
Abdominal pain	25 (21.0)	3	22	
Other (perforation, peritonitis)	1 (0.1)	1	0	
Asymptomatic patients at diagnosis, incidental findings on CT scan	35 (29.4)	2 (11.1)	33 (32.7)	
Other symptoms at diagnosis, not specific for GIST	32 (26.9)	4 (22.2)	28 (27.7)	
GIST characteristics				
Median number of GIST at diagnosis, *n* (range)	2 (1-40)	5.5 (1-40)	3.5 (1-20)	0.67
Largest GIST location				0.71
Stomach	13 (11)	2 (11)	11 (11)	
Small bowel (duodenum, jejunum, ileum)	102 (86)	15 (83)	87 (86)	
Of which duodenum	31 (26)	3 (17)	28 (28)	
Indeterminate	4 (3)	1 (6)	3 (3)	
Median largest GIST size, mm (range)	45.0 (3-220)	71.5 (35-140)	48.4 (3-220)	**0.001**
Largest GIST size, mm				**0.003**
0-30	35 (29.4)	0	35	
30-50	32 (26.9)	5	27	
50-100	47 (39.5)	11	36	
>100	5 (4.2)	2	3	
Median mitotic index/5 mm^2^ of largest GIST (range)	2 (0-75)	7 (0-40)	6 (0-75)	0.16
Mitotic index/5 mm^2^				**0.04**
0-5	89 (79.5)	11	78	
5-10	13 (11.6)	5	8	
>10	10 (8.9)	2	8	
NA	7	—	—	
Tumor rupture				0.11
Yes	4 (3.6)	2 (11.8)	2 (2.0)	
No	107 (96.4)	15 (88.2)	92 (98.0)	
Median Ki67, % (range)	3 (0.9-20.0)	6.6 (1.9-20.0)	3.8 (0.9-20.0)	0.32
NA	60	—	—	
Molecular analysis				**0.04**
*KIT/PDGFRA* mutation	5 (5.1)	3 (17.7)	2 (2.4)	
*NF1* or no *KIT/PDGFRA* mutation	93 (94.9)	14 (82.3)	79 (97.6)	
Risk of recurrence according to Joensuu				**0.01**
Very low	14 (12.3)	0	14 (14.6)	
Low	36 (31.6)	2 (11.1)	34 (35.4)	
Intermediate	4 (3.5)	0	4 (4.2)	
High	60 (52.6)	16 (88.9)	44 (45.8)	
NA	5	—	—	
Risk of recurrence according to Miettinen				**0.009**
Null	13 (13.0)	0	13 (15.5)	
Very low	4 (4.0)	1 (6.7)	3 (3.5)	
Low	34 (34.0)	1 (6.7)	33 (39.0)	
Intermediate	29 (29.0)	7 (46.6)	22 (25.0)	
High	20 (20.0)	6 (40.0)	14 (17.0)	
NA	19	—	—	
Adjuvant				**<0.001**
Yes	21 (17.6)	11 (50.0)	11 (50.0)	
No	93 (78.2)	7 (7.2)	90 (92.8)	
Prognosis				
Death	18	10	8	
Median age of death, year (range)	67 (43-80)	—	—	
Death causes				**0.046**
Related to GIST evolution	8 (50.0)	7	1	
Related to NF1 evolution	2 (12.5)	0	2	
Other causes	6 (37.5)	2	4	
NA	2	—	—	

Bold indicates *P* < 0.05.

GIST, gastrointestinal stromal tumor; NA, not available; NF1, neurofibromatosis type 1.

The median age at diagnosis was 53 years (range 20-78 years) and patients were mainly women (61.3%). The diagnosis of GIST was based on a complication in 43.7% (among them: 21.0% of pain, 16.8% hemorrhage, 5.0% occlusion, and one case of peritonitis); GIST were incidental during follow-up of NF1 disease in 29.4%; and nonspecific symptoms (such as anemia, hypertension or headache) led to diagnosis in 26.9% of cases. Median follow-up of the cohort was 6.0 years. Eighteen patients died, 44.4% from the GIST, 11.2% from another NF1-related cancer (mainly nervous system tumors) and 33.3% without obvious relationship to NF1 or GIST. The median age at death was 67.0 years (range 43-80 years).

### GIST characteristics

NF1-GIST characteristics are detailed in Table 1. They were mostly located in the small bowel (60.0% in the jejuno-ileum and 26.0% in the duodenum); a few were located in the stomach (11.0%), and 3.0% were of unknown location (missing data). Multiple GIST were present in 58.8% of cases and the median number of simultaneous GIST was 2 (range 1-40).

The median tumor size of the largest GIST at diagnosis was 45.0 mm (range 3-220 mm), 29.4% of GIST had a size ≤30 mm and 43.7% a size >50 mm. Median mitotic count was 2/5 mm^2^ (range 0-75/5 mm^2^), and 79.5% had a mitotic count of ≤5/5 mm^2^. Tumor rupture was observed in 3.6% of patients. Ki67 was available for 60% of patients and the median value was 3.0% (range 0.9%-20.0%).

There was no difference between size and mitotic count regarding location: 45.0 mm versus 47.0 mm versus 54.0 mm (*P* = 0.26) and 2 mit/5 mm^2^ versus 4.5 mit/5 mm^2^ versus 2 mit/5 mm^2^ (*P* = 0.20) for small bowel versus gastric versus unknown primary GISTs, respectively.

Analysis of somatic mutations was available for 98 patients: 95.0% did not harbor any *KIT* or *PDGFRA* mutation. A *NF1* somatic mutation was identified in only six GISTs (only one gastric GIST), but the *NF1* gene was not systematically tested for all patients. When tested, however, a *NF1* mutation was systematically found (all different, involving several exons). Among the *PDGFRA*-mutated GISTs (3.0%), one mutation involved exon 18 (p.Arg841_Ile843del), the two others being unspecified. For *KIT*-mutated GISTs (2.0%), one involved exon 12 (p.Thr607Asn) and the other one involved exon 13. No other mutation was observed (*RAS*, *BRAF* or *SDH* mutations). There was no difference in mutational status (*KIT/PDGFRA* versus no *KIT/PDGFRA* mutation) regarding the location of the GIST (*P* = 0.62).

**Figure 1:**
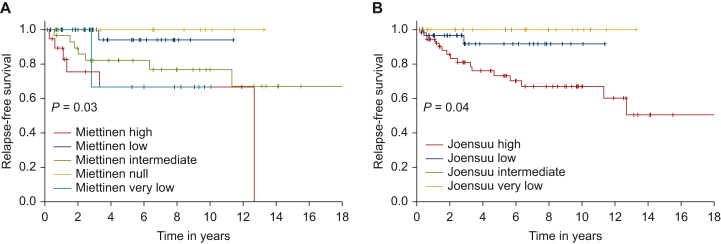
Relapse-free survival of neurofibromatosis type 1 gastrointestinal stromal tumors according to (A) Miettinen’s Armed Force Institute of Pathology (AFIP) classification and (B) Joensuu’s modified National Institutes of Health (NIH) classification.

Miettinen and Joensuu classifications were available for 84.0% and 95.8% of GISTs, respectively. According to Miettinen’s classification, NF1-GISTs were at high, intermediate, low, very low and null risk for 20.0%, 29.0%, 34.0%, 4.0% and 13.0%, respectively. According to Joensuu’s classification, the risk was 52.6%, 3.5%, 31.6% and 12.3% for high, intermediate, low and very low, respectively. RFS of the risk groups was different according to Miettinen’s (*P* = 0.03) and Joensuu’s (*P* = 0.04) classifications (Figure 1).

### Survival data and factors associated with relapse

Median OS (mOS) of the cohort was not reached. The 5-year and 10-year OS were 89.0% and 81.4%, respectively. Relapse occurred in 18 patients (15.1%); metastatic site was the peritoneum in 77.8% and the liver in 38.9% of cases. mRFS was not reached; the 5-year RFS was 83.8% (95% CI 73.6% to 89.7%), the 10-year RFS was 81.2% (95% CI 70.6% to 88.3%), and the 15-year RFS was 65.1% (95% CI 39.6% to 82.0%). For patients with GIST recurrence, mOS from the date of diagnosis was 13.1 years, and 5.3 years from the date of relapse, with a 10-year OS of 50.0%. In comparison, mOS of patients without GIST recurrence was not reached and the 10-year OS was 88.2%.

In univariate analysis (Table 2), factors associated with relapse were age >65 years (HR 3.3, 95% CI 1.3-8.3, *P* = 0.04), revelation of the GIST through a complication (HR 3.6, 95% CI 0.8-16.1, *P* = 0.10), tumor size >37 mm (HR 6.5, 95% CI 0.8-50.4, *P* = 0.07), and mitotic count >5 mit/5 mm^2^ (HR 4.9, 95% CI 1.8-13.2, *P* < 0.01). The cut-off of size was chosen after multiple statistic tests. There were not enough events to analyze tumor rupture and mutational status. Risk of relapse for *KIT/PDGFRA*-mutated GISTs, however, was higher than non-*KIT/PDGFRA*-mutated GISTs (60% versus 18%, *P* = 0.04).

**Table 2:** Univariate and multivariate analysis of factors associated with relapse

	RFS Univariate analysis				RFS Multivariate analysis		
	*n* (event)	Hazard ratio	95% CI	*P*	Hazard ratio	95% CI	*P*
Age, years				**0.04** **0.01**			
Risk for each 1-unit increase	119	1.04	1-1.1				
≤65	85	1	1		1		
>65	33	3.26	1.3-8.3		2.75	0.964-7.83	**0.06**
Sex							
Female		1					
Male		1.03	0.4-2.7	0.95			
Revelation mode							
Follow-up of NF1	35	1			1		
Complication	51	3.55	0.8-16.1	**0.10**	2.7	0.6-12.7	0.2
Other	32	2.05	0.4-11.3	0.41	1.0	0.2-6.6	0.99
Size (mm)	41 77				1 3.6	0.8-16.6	**0.10**
Risk for each 1-unit increase		1	0.9-1.0	0.44			
≤37		1					
>37		6.51	0.84-50.4	**0.07**			
≤20		10^−8^	0-+∞				
21-50		1					
50-100		2.06	0.71-6.0	0.18			
>100		3.3	0.6-17.2	0.17			
Mitotic count (*n*/5 mm^2^)				**<0.01** **<0.01**			
Risk for each 1-unit increase		1.15	1.0-1.3				
≤5	88	1			1		
>6	23	4.89	1.8-13.2		3.9	1.3-11.6	**0.01**
Rupture							
No	107	NA		0.11			
Yes	4						
Ki67 (%)							
0-5							
5-10							
>10							
Mutation							
Non-*KIT*/non-*PDGFRA* GIST	92	NA		0.03			
*KIT/PDGFRA* GIST	5						
Tumor growth rate				0.9			
≤0	17						
>0	27						
RECKGIST score							
RECKGIST A	35	10^−8^	0-+∞	0.99			
RECKGIST B	60	1					
RECKGIST C	20	4.18	1.54-11.38	**<0.01**			

Bold indicates *P* < 0.05.

CI, confidence interval; GIST, gastrointestinal stromal tumor; NA, not available; NF1, neurofibromatosis type 1; RFS, recurrence-free survival.

In multivariate analysis (Table 2 and Supplementary Figure S1, available at https://doi.org/10.1016/j.esmoop.2025.104477), the only remaining factor associated with relapse was mitotic count >5 mit/5 mm^2^ (HR 3.9, 95% CI 1.3-11.6, *P* = 0.01).

### Development of a new prognostic score for NF1-GIST

RFS was significantly different between the risk subgroups of the Miettinen and the Joensuu classifications, but the classifications did not discriminate high-risk NF1-GISTs well (Figure 1). Therefore we aimed to develop another prognostic model from our cohort.

**Figure 2:**
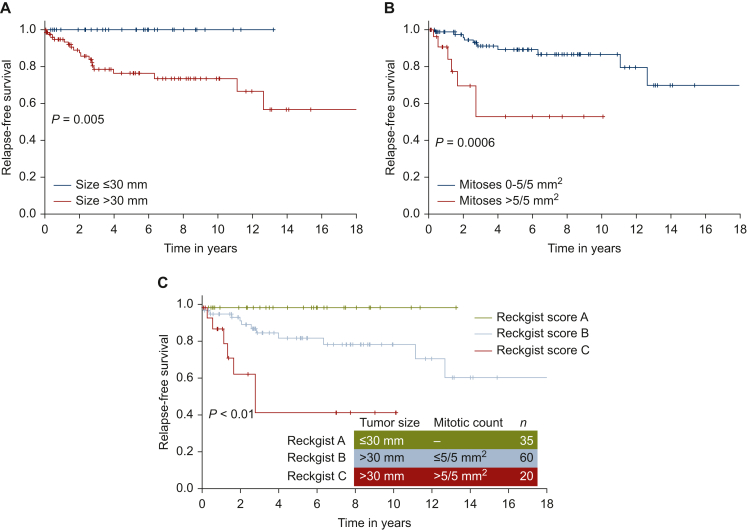
Relapse-free survival of neurofibromatosis type 1 gastrointestinal stromal tumors according to (A) tumor size, (B) mitotic count and (C) RECKGIST classification.

For NF1-GIST ≤30 mm (*n* = 35), no recurrence was observed after surgery (Figure 2A), despite three tumors with a high mitotic count (two with 8 mit/5 mm^2^ and one with 10 mit/5 mm^2^). Several values of mitotic count were tested in tumors >30 mm (*n* = 80; data not shown) and the best value to predict relapse was 5 mit/5 mm^2^ in this subgroup (Figure 2B): 60 patients had a mitotic count ≤5/5 mm^2^ and 20 had a value >5 mit/5 mm^2^ (*P* < 0.01).

Based on these findings, we built the RECKGIST score as follows: score A was classified as tumor size ≤30 mm irrespective of the mitosis number, score B as tumor size >30 mm and a mitotic count of between 0 and 5 included, and score C as a tumor size >30 mm and a mitotic count >5 mit/5 mm^2^. Full data (size, mitotic count) were available for 115 patients. Thirty-five patients were in the RECKGIST A group, 60 in the RECKGIST B group and 20 in the RECKGIST C group.

The 5-year RFS and 10-year RFS were 100% for the RECKGIST A subgroup, 83.3% and 79.8% for the RECKGIST B subgroup, and 54.0% and 43.2% for the RECKGIST C subgroup, respectively (Figure 2C). Difference in RFS was highly significant between the three groups (*P* < 0.01), and the HR between RECKGIST B and RECKGIST C groups was 3.67 (95% CI 1.38-9.76, *P* < 0.01). As there was no relapse in the RECKGIST A subgroup, HR could not be calculated. Interestingly, the RECKGIST score better predicted RFS than the Miettinen (*P* = 0.03) and Joensuu classifications (*P* = 0.04).

### Impact of adjuvant imatinib treatment on risk of recurrence in operated NF1-GISTs

Among all patients undergoing surgery, 17.6% (*n* = 21) were treated with adjuvant imatinib for at least 3 months. A few patients (4.2%, *n* = 5) had exclusive neoadjuvant treatment and were excluded from this analysis, including one patient who had neoadjuvant sunitinib. Overall, 114 patients were selected for the adjuvant treatment analysis: 21 in the AG and 93 in the SG (Supplementary Table S1, available at https://doi.org/10.1016/j.esmoop.2025.104477). In the AG, NF1-GISTs were at high risk of relapse (Joensuu high risk: 90.0%, Miettinen high risk: 64.7%, RECKGIST C: 40.0%). Median duration of imatinib was 22.5 months (interquartile range 6.5-36.0 months). Four patients had a duration of imatinib ≤6 months, 7 patients had at least 1 year and 8 patients had 3 years of imatinib treatment.

**Figure 3:**
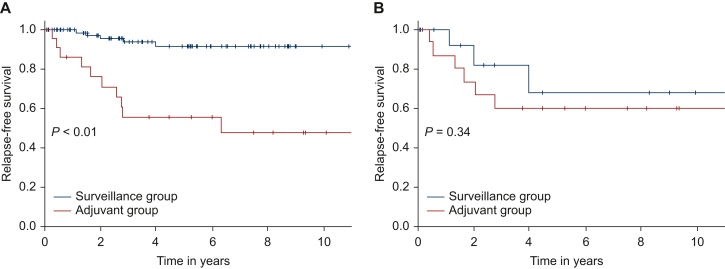
Relapse-free survival of neurofibromatosis type 1 gastrointestinal stromal tumors in the adjuvant group and in the surveillance group (A) before matching and (B) after matching.

Half of the AG (52.3%) experienced recurrence, compared with 7.2% of the SG (*P* < 0.001), and 10-year RFS was 47.9% (95% CI 23.8% to 68.6%) in the AG versus 91.7% (95% CI 80.8% to 96.5%) in the SG (Figure 3A; log-rank, *P* < 0.001). Before matching, both groups were similar for sex, age at diagnosis, cause of death, revelation mode, location of the GIST, tumor rupture, and mutational status. There was a difference for size (median: 68 mm in the AG versus 48 mm in the SG, *P* = 0.02), mitotic count (median: 7 mit/5 mm^2^ versus 2 mit/5 mm^2^, *P* = 0.004), Miettinen score ‘High’ (64.7% versus 10.3%, *P* < 0.001), Joensuu score ‘High’ (90.0% versus 44.3%, *P* = 0.003), and RECKGIST score C (40.0% versus 9.9%, *P* = 0.001), respectively.

The IPTW matching method did not properly match patients, as the AG remained of worse prognosis (data not shown). In the RECKGIST C group, eight received imatinib treatment and five relapsed; conversely one out of nine patients undergoing surveillance experienced recurrence. Best 1 : 1 matching was only possible for 15 patients from each group. After matching (Supplementary Table S1, available at https://doi.org/10.1016/j.esmoop.2025.104477), there was no difference for year of diagnosis (*P* = 0.14), location (gastric 13.3% versus 20%, *P* = 0.99), mutational status (*KIT/PDGFRΑ* 0% versus 7%, *P* = 0.48), largest size (76 mm versus 53 mm, *P* = 0.42), mitotic count (8/5 mm^2^ versus 5/5 mm^2^, *P* = 0.86) and RECKGIST score C (31% versus 20%, *P* = 0.69) for AG and SG, respectively. RFS was similar between both groups (Figure 3B; log-rank, *P* = 0.34), and 10-year RFS was 60.0% (95% CI 31.8% to 79.7%) and 67.9% (95% CI 28.2% to 88.8%), respectively.

## Discussion

To our knowledge, this is one of the largest multicentric cohorts of NF1-GISTs for which clinical and histological characteristics, as well as risk factors for relapse and survival, have been evaluated. The baseline characteristics of our population were very similar to those previously published^10^, ^11^, ^12^: an early onset at diagnosis, female predominance, a majority of lesions located in the small bowel and the presence of multiple GISTs. The female predominance is remarkable considering the 1 : 1 sex ratio in sporadic GISTs and in with Von Recklinghausen disease, distributed evenly between men and women. Data from previous published studies are conflicting, with this difference found in Miettinen’s^11^ cohort but not in Salvi’s study^10^, and may be due to a rather small sample size.

Surprisingly, gastric GISTs comprised 11.0% of cases, there were no colorectal GISTs, and 5.1% of NF1-GISTs harbored a *KIT* or *PDGFRA* mutation. This was also described by Miettinen et al., and highlights the possibility that NF1 patients experience sporadic GISTs not linked to constitutional *NF1* mutation. This phenomenon has already been described in other genetic diseases such as Lynch syndrome, in which proficient mismatch repair (MMR) colorectal carcinoma may appear instead of deficient MMR carcinomas.^17^ These *KIT/PDGFRA-*mutated GISTs are indeed different, as they seem to have a worse prognosis with a relapse risk three times higher than non-*KIT/PDGFRA*-mutated NF1-GISTs (60% versus 18%). These findings warrant the determination of *KIT/PDGFRA* status in NF1-GISTs and adjuvant imatinib treatment must be discussed at a multidisciplinary tumor board regarding high-risk *KIT* or non-p.D842V *PDGFRA*-mutated NF1-GISTs.

Although often described as benign, NF1-GISTs had a recurrence rate of 15.1% and a 15-year RFS of 65.1%. In contrast, sporadic GISTs have a 15-year RFS of ∼60%. For patients who relapsed, mOS from date of surgery of primary tumor was similar: 10.3 years in NF1-GISTs compared with 12.4 years in sporadic GISTs.^14^ This study sheds new light on relapse risk in NF1-GISTs. Firstly, we chose relapse over death to better describe this rare disease, as NF1 patients may develop and die from other malignant tumors. Secondly, known risk factors associated with relapse for sporadic GISTs (size, mitotic count, location, tumor rupture, mutation) have never been studied in the NF1-GIST subpopulation. For sporadic tumors, 10-year RFS for GIST >2 cm was 85%, and 78% for GIST from 2 to 5 cm.^14^ We demonstrate the excellent prognosis of NF1-GISTs ≤3 cm without any recurrence, even for the three patients with high mitotic count. For NF1-GISTs >3 cm, the 5 mit/5 mm^2^ cut-off was highly predictive of relapse, with a 10-year RFS of 79.8% for patients below and 43.2% for patients above this threshold. Moreover, this value was the only factor associated with relapse in the multivariate analysis, and is consistent with the high predictive value of mitotic count in predicting recurrence in GISTs. We could not properly demonstrate the role of tumor rupture and mutational status (numbers in both groups were too small), even though there is a probability that these two factors have an impact on recurrence probability.

Regarding usual risk classifications, NF1-GISTs classified as Miettinen (20.0% of cases) or Joensuu (52.6% of cases) ‘high risk’ had a 10-year RFS of ∼60%. Miettinen ‘intermediate risk’ group had a similar 10-year RFS of 75%, and ‘low’, ‘very low’ and ‘null risk’ groups were not clearly separated. For the Joensuu classification applied to NF1-GISTs, only the ‘high risk’ group was distinct from the others. The RECKGIST score developed in this study seems to better classify patients with this particular type of GIST, but it needs validation in an external cohort. Moreover, these results may be enhanced in the future by an update, as follow-up (6 years) may have not been long enough to identify all patients experiencing relapse. However, we have observed that there is a plateau phase after 12 years, suggesting a low late recurrence rate.

Finally, there was no evidence for better survival with adjuvant therapy. Of note, 17.6% of patients received adjuvant treatment. This percentage is high, reflecting outdated management of GIST that is no longer recommended. These data are consistent with the low efficacy of imatinib in NF1-GISTs in the metastatic setting.^18^ Matching was difficult, as a majority of GIST patients on imatinib had poorer prognostic factors than those with GIST undergoing surveillance without further adjuvant treatment. The data may also reflect the different management of these GISTs over time: early patients were operated on for larger tumors with higher mitotic count (data not shown), whereas later patients were managed at an earlier stage. Even if the small numbers in the AG and the SG groups may explain the absence of significant differences in baseline characteristics, both groups after matching had a similar prognosis. Based on these data, it appears that a randomized control study to demonstrate the utility of imatinib in high-risk NF1-GISTs (group RECKGIST C) would be time-consuming and futile in this rare disease. A predictable difficult accrual (recruitment) in a study that will be particularly hard to finish.

The main strengths of this cohort, besides its originality, are the size and the long follow-up. Another strength is the quality of the data, which were collected nationwide from the personal files of consecutive patients, allowing a small amount of missing data for key prognostic factors [100.0% for size and number of GISTs (*n* = 119), 95.8% for mitotic count (*n* = 114), 82.4% for *KIT/PDGFRA* mutational status (*n* = 98)]. Baseline characteristics are in line with those previously published and our population seems representative of NF1-GISTs.

The main limitations of the study are its retrospective design, which may have led to confusion or data collection bias. In some subgroup analyses (e.g. for the risk of rupture or mutational status influencing relapse or the study of adjuvant therapy), the number of patients may have been too small to detect a significant difference. On a molecular level, extended NGS mutational status, including *NF1* gene, was lacking. However, this testing is not recommended by the French^2^ or the ESMO guidelines^19^ and is still not routinely carried out, even in tertiary centers. Moreover, we can assume that almost every GIST of our cohort harbored an *NF1* mutation, as all patients had Von Recklinghausen disease and therefore a constitutional *NF1* mutation. Finally, no difference in term of relapse had been identified between the 6 GISTs with an *NF1* mutation and the 108 non-*KIT/PDGFRA*-mutated NF1-GISTs (data not shown; log-rank, *P* = 0.45). A complete molecular characterization could be useful to better define the prognosis of *NF1* mutations or the occurrence of partner mutations.

An international prospective cohort with fewer missing data would be useful to strengthen these findings. Finally, this study lacks a validation cohort for the RECKGIST score. As NF1-GISTs are very rare, further external collaboration with other institutions outside France could be helpful to better characterize them.

### Conclusion

NF1-GISTs arise mostly in the small bowel, but 11% are located in the stomach. Five percent harbor a *KIT* or *PDGFRA* mutation. In GISTs <3 cm, prognosis is excellent without relapse and may define a new cut-off for surgery in cases of asymptomatic tumor. There appears to be no benefit from adjuvant treatment even if the level of evidence in this study is low, except for high-risk *KIT* or non-p.D842V *PDGFRA*-mutated GISTs, which may benefit from adjuvant therapy. The RECKGIST score classifies NF1-GISTs more accurately than the usual prognostic classifications. Further studies are warranted to better characterize these rare tumors.

## Disclosure

**MB**: Deciphera, Bayer, Boehringer, Pharmamar. **BV**: consultant fees from Lilly, Pfizer, netcancer, Pierre Fabre, Seagen, Daiichi Sankyo, Gilead, Novartis, Merck, Sharp, Dohme (MSD), AstraZeneca (AZ), Owkins, Boehringer Ingelheim. Received travel expenses from Lilly, Novartis, Pfizer, Accord Healthcare, Amgen, AZ, Daiichi Sankyo. **AG**: Bayer, Roche, Merck. **SW**: Deciphera, Pharmamar, Boehringer, AZ. **SM**: travel grants from Roche, AZ, MSD, Servier. **DT**: Amgen, Roche, Merck Serono, MSD, Bristol Myers Squibb, Pierre Fabre, Servier, Takeda, AZ. **OB**: Amgen, Bayer, Deciphera Bayer Boehringer, Merck, MSD, Pierre Fabre, Servier, Takeda. **VH**: AAA, Amgen, Esteve, Ipsen, Deciphera, Merck, Pierre Fabre, Servier. All other authors have declared no conflicts of interest.
