# Supplementary material for: Clinical description and development of a prognostic score for neurofibromatosis type 1 (NF1)-associated GISTs: a retrospective study from the NETSARC+
Source: ESMO Open. 2025 Mar 4;10(3):104477. doi: 10.1016/j.esmoop.2025.104477 (PMC11928958; doi:10.1016/j.esmoop.2025.104477)

**Supplementary Figure S1:** Forrest plot diagram representing the results of the multivariate analysis of factors associated with relapse


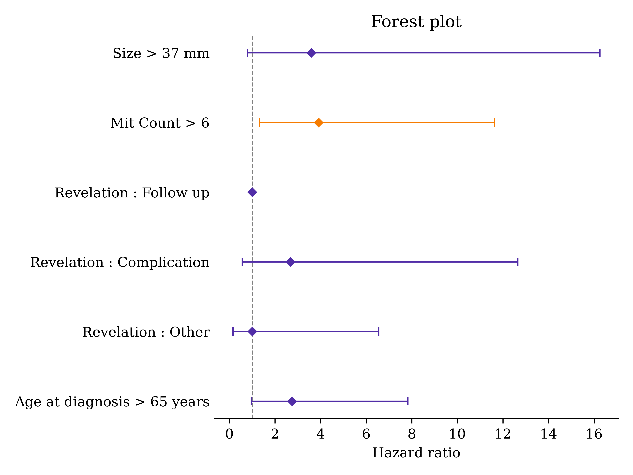

Supplement: Supplementary Figure S1 [file mmc1.docx]
