# Supplementary material for: Clinical description and development of a prognostic score for neurofibromatosis type 1 (NF1)-associated GISTs: a retrospective study from the NETSARC+
Source: ESMO Open. 2025 Mar 4;10(3):104477. doi: 10.1016/j.esmoop.2025.104477 (PMC11928958; doi:10.1016/j.esmoop.2025.104477)
Supplement: Supplementary Table S1 [file mmc2.docx]

**Supplementary Table S1:** Main characteristics of the adjuvant group (AG) and the surveillance group (SG) before and after matching

|  | **Before matching** | |  | **After matching** | |  |
| --- | --- | --- | --- | --- | --- | --- |
|  | **Adjuvant Group** | **Surveillance Group** | **p** | **Adjuvant Group** | **Surveillance Group** | **p** |
| **Total** | 21 | 93 | - | 15 | 15 |  |
|  |  |  |  |  |  |  |
| **Gender**  Female  Male | 14 (66.7%)  7 (33.3%) | 57 (61.3%)  36 (38.7%) | 0.80 | 9 (60.0%)  6 (40.0%) | 6 (40.0%)  9 (60.0%) | 0.47 |
| **Age at diagnosis (years)** | 55 (22-75) | 52 (20-78) | 0.2 | 56 (22-75) | 54 (23-73) | 0.70 |
| **Revelation mode**  Follow-up of NF1  Complication  Other | 5 (23.8%)  12 (57.1%)  4 (19.1%) | 28 (30.1%)  39 (41.9%)  26 (28.0%) | 0.44 | 2 (13.3%)  9 (60.0%)  4 (26.7%) | 2 (13.3%)  9 (60.0%)  4 (26.7%) | 0.99 |
| **GIST location**  Stomach  Duodenum  Small bowel  Unknown | 1 (4.8%)  5 (23.7%)  14 (66.7%)  1 (4.8%) | 11 (11.8%)  24 (25.8%)  55 (59.2%)  3 (3.2%) | 0.76 | 2 (13.3%)  4 (26.7%)  8 (53.3%)  1 (6.7%) | 3 (20.0%)  5 (33.33%)  7 (46.67%)  0 (0.0%) | 0.99 |
| **Size (mm)** | 48 (3-210) | 70 (18-220) | **0.02** | 75 (18-220) | 53 (3-100) | 0.42 |
| **Mitoses** | 7 (1-40) | 2 (0-75) | **0.004** | 8 (1-40) | 6 (0-16) | 0.86 |
| **Rupture**  Yes  No | 2 (10.5%)  17 (89.5%) | 2 (2.3%)  86 (97.7%) | 0.14 | 2 (10.0%)  18 (90.0%) | 2 (2.2%)  89 (97.8%) | 0.15 |
|  |  |  |  |  |  |  |
| **GIST Mutation**  Non-*KIT/PDGFRA*  *KIT/PDGFRA* | 17 (89.5%)  2 (10.6%) | 72 (96.0%)  3 (4.0%) | 0.29 | 14 (100.0%)  0 (0.0%) | 12 (92.3%)  1 (7.8%) | 0.48 |
|  |  |  |  |  |  |  |
| **Cause of death**  GIST  NF1 neoplasia other than GIST  Other | 3 (42.9%)  0 (0.0%)  4 (57.1%) | 8 (53.3%)  2 (13.4%)  5 (33.3%) | 0.54 | 3 (60.0%)  1 (20.0%)  1 (20.0%) | 3 (42.9%)  0 (0.0%)  4 (57.1%) | 0.37 |
|  |  |  |  |  |  |  |
| **Miettinen Classification**  Null  Very Low  Low  Intermediate  High | 1 (5.9%)  0 (0.0%)  1 (5.9%)  4 (23.5%)  11 (64.7%) | 12 (15.4%)  4 (5.1%)  31 (39.7%)  23 (29.5%)  8 (10.3%) | **<0.001** | 1 (9.1%)  0 (0.0%)  0 (0.0%)  4 (36.4%)  6 (54.5%) | 1 (9.1%)  0 (0.0%)  2 (18.1%)  4 (36.4%)  4 (36.4%) | 0.77 |
|  |  |  |  |  |  |  |
| **Joensuu Classification**  Very low  Low  Intermediate  High | 1 (5.0%)  1 (5.0%)  0 (0.0%)  18 (90.0%) | 13 (14.8%)  32 (36.4%)  4 (4.5%)  39 (44.3%) | **0.003** | 1 (7.1%)  1 (7.1%)  0 (0.0%)  12 (85.8%) | 2 (14.4%)  2 (14.4%)  1 (7.0%)  9 (64.2%) | 0.53 |
| **RECKGIST classification**  A  B  C | 2 (10.0%)  10 (50.0%)  8 (40.0%) | 32 (35.1%)  50 (55.0%)  9 (9.9%) | **0.001** | 2 (14.3%)  7 (50.0%)  5 (35.7%) | 3 (20.0%)  9 (60.0%)  3 (20.0%) | 0.69 |
